# Supplementary material for: Development and Validation of Early Alert Model for Diabetes Mellitus–Tuberculosis Comorbidity
Source: Microorganisms. 2025 Apr 16;13(4):919. doi: 10.3390/microorganisms13040919 (PMC12029158; doi:10.3390/microorganisms13040919)
Supplement: Supplementary file 1 [file microorganisms-3508458-Supplementary.pdf › microorganisms-3508458-Supplementary.pdf]

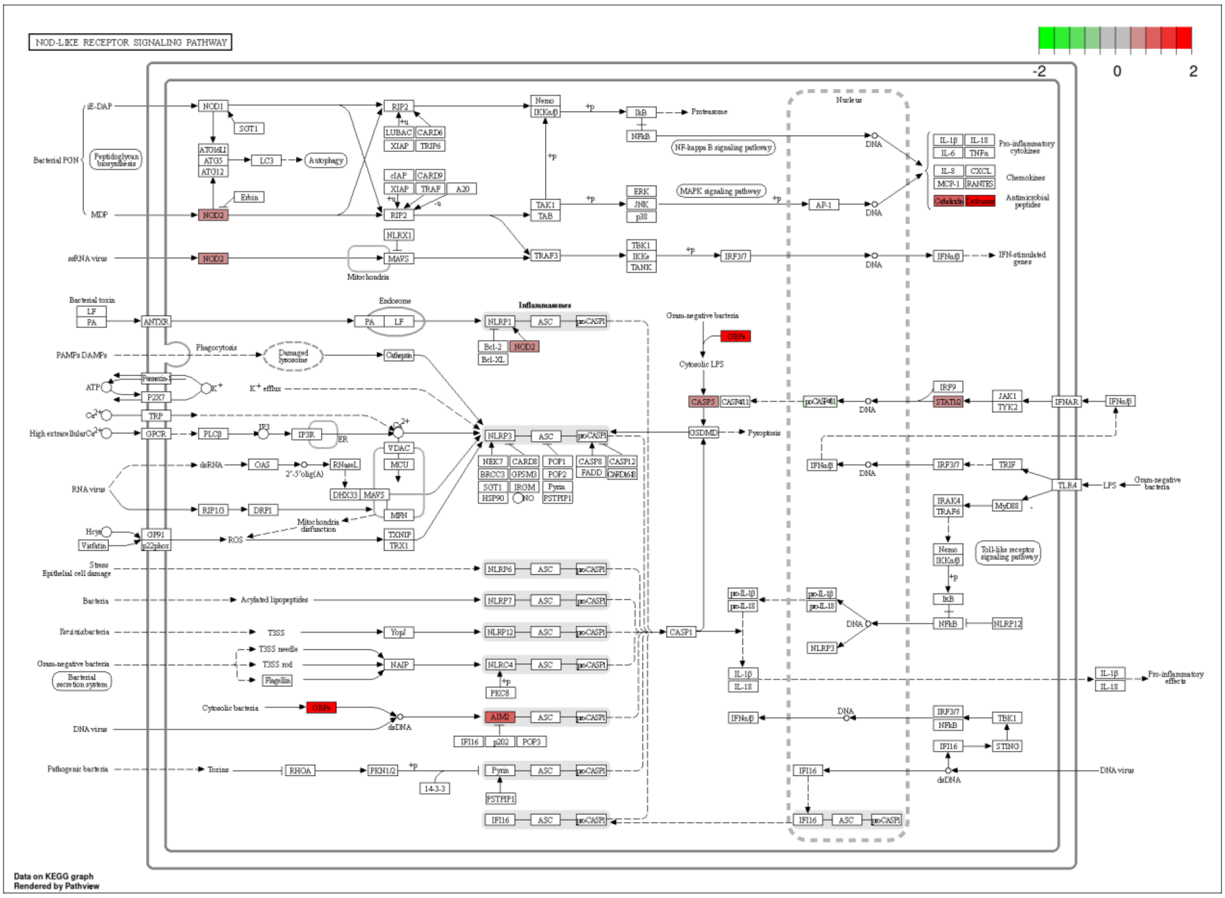

Figure S1. NOD-like receptor signal-ing pathway.
